# Development and validation of a diagnostic nomogram based on peripheral blood composite inflammatory markers for complicated acute appendicitis: a retrospective study

**DOI:** 10.3389/fsurg.2026.1784195

**Published:** 2026-04-30

**Authors:** Menghao Li, Canjun Luo, Jimiao Qiu, Jiawei Luo, Li Li, Chengzhong Zhou, Gang Sun, Mengyan Wang

**Affiliations:** 1 Department of General Surgery, the 906th Hospital of Joint Logistic Support Force of PLA, Ningbo, China; 2 Department of Liver Diseases and Digestive Interventional Radiology, Digestive Diseases Hospital, Xi'an International Medical Center Hospital, Northwest University, Xi'an, China; 3 Department of Radiation Oncology, the 906th Hospital of Joint Logistic Support Force of PLA, Ningbo, China

**Keywords:** complicated acute appendicitis, composite inflammatory markers, diagnostic value, nomogram, peripheral blood

## Abstract

**Background:**

Accurate distinction between complicated acute appendicitis (CAA) and simple acute appendicitis (SAA) is crucial for guiding treatment decisions. This study aimed to systematically evaluate the diagnostic value of peripheral blood inflammatory composite markers and develop a predictive model for CAA.

**Methods:**

In this retrospective study, 533 patients with acute appendicitis (429 in the training set and 104 in the validation set) admitted between January 2018 and December 2024 were enrolled. Various peripheral blood composite inflammatory markers were calculated. Multivariate stepwise logistic regression identified independent predictors. Based on these predictors, a diagnostic nomogram was constructed and its performance was evaluated using receiver operating characteristic (ROC) curve analysis, calibration curves, and decision curve analysis (DCA).

**Results:**

The Systemic Inflammation Response Index (SIRI, OR = 2.972, 95% CI: 1.722–5.187, P < 0.001), Neutrophil-to-Albumin Ratio (NAR, OR = 3.099, 95% CI: 1.693–5.723, *P* < 0.001), and Prognostic Nutritional Index (PNI, OR = 0.553, 95% CI: 0.309–0.969, *P* = 0.041) were identified as independent predictors. The areas under the ROC curve (AUC) for SIRI, NAR, and PNI were 0.739, 0.742, and 0.535, respectively. The nomogram model incorporating these three markers showed an AUC of 0.758 (95% CI: 0.714–0.803) in the training set and 0.756 (95% CI: 0.656–0.857) in the validation set. Calibration curves indicated good agreement between predicted and observed outcomes (mean absolute error [MAE] = 0.022 and 0.025, respectively), and DCA confirmed the clinical utility of the model across a range of threshold probabilities in both cohorts.

**Conclusion:**

This study demonstrates that SIRI, NAR, and PNI are independently associated with acute appendicitis severity. The developed nomogram, integrating these three markers, offers a simple, cost-effective tool with good diagnostic performance for distinguishing CAA from SAA, potentially aiding in timely clinical decision-making and individualized treatment planning.

## Introduction

Acute appendicitis is one of the most common surgical acute abdominal conditions, with its global occurrence rate having increased by 63.5% over the past decade (1). Acute appendicitis can be further classified into simple acute appendicitis and complicated acute appendicitis. The occurrence rate of complicated appendicitis is as high as 28%–29%, imposing a considerable burden on healthcare systems (2, 3). Currently, the standard treatment for acute appendicitis is surgical resection via appendectomy (3). However, a comparative study published in The Lancet on antibiotic therapy vs. appendectomy for acute appendicitis demonstrated that antibiotic treatment is non-inferior to appendectomy for patients with uncomplicated acute appendicitis (4). Meanwhile, with the emergence of endoscopic retrograde appendicitis therapy, treatment options for acute appendicitis have significantly expanded (5). Additionally, amid increasing social stress and an accelerated pace of life, a growing number of young patients with appendicitis prefer conservative treatment. However, conservative management for complicated appendicitis is often less effective and carries a higher risk of severe complications, leading to prolonged hospitalization, increased mortality, and elevated medical costs (6, 7). Therefore, accurate classification of acute appendicitis subtypes is crucial for guiding clinical decision-making. Several scoring systems have been developed to aid in the diagnosis of acute appendicitis (8), nevertheless, these tools are limited in their ability to effectively predict disease severity. Composite inflammatory markers derived from peripheral blood tests are characterized by their simplicity and cost-effectiveness and are widely used for disease diagnosis and prognosis. Inflammatory indices such as the neutrophil-to-albumin ratio (NAR) (9), Systemic Inflammation Response Index (SIRI) (10), platelet-to-lymphocyte ratio (PLR) (11) and neutrophil-to-lymphocyte ratio (NLR) (12) have been shown in various studies to possess certain predictive value for the severity of appendicitis. Nevertheless, many of these studies suffer from the limitation of not comprehensively including a wide range of inflammatory markers. Thus, this study aims to systematically investigate key predictors of complicated acute appendicitis by incorporating multiple peripheral blood-derived composite inflammatory markers and to develop a diagnostic nomogram model. This approach is expected to provide a clinically practical solution for the precise stratification of acute appendicitis severity.

## Materials and methods

### Study subjects

This retrospective study enrolled patients who were treated for acute appendicitis at the 906th Hospital of Joint Logistics Support Force of Chinese People's Liberation Army (PLA) between January 2018 and December 2024 (Full dataset). Among them, patients from January 2018 to December 2022 were included in the training set, and patients from January 2023 to December 2024 were assigned to the validation set. All subjects met the following inclusion and exclusion criteria. Inclusion criteria: 1) Definitive diagnosis of acute appendicitis confirmed by imaging examinations (including appendiceal ultrasound and abdominal computed tomography) combined with laboratory test results; 2) Age ranging from 18 to 60 years; 3) No history of digestive tract diseases, e.g., inflammatory bowel disease, gastrointestinal tumors, irritable bowel syndrome; 4) No use of antibiotics or hormonal medications within the 3 months prior to enrollment; 5) Good compliance and cooperation with diagnosis and treatment. Exclusion criteria: 1) Refusal to undergo surgical appendectomy; 2) Pregnancy or lactation; 3) Incomplete clinical data. Based on the final postoperative pathological reports, patients were divided into two groups: the simple acute appendicitis (SAA) group, pathologically diagnosed with simple acute appendicitis, and the complicated acute appendicitis (CAA) group, pathologically diagnosed with acute suppurative appendicitis, gangrenous appendicitis, or appendiceal perforation.

### Data collection

General data collected included age, gender, smoking history (defined as smoking >1 cigarette per day for >6 months), and alcohol consumption history (defined as weekly alcohol intake >10 g for >1 year). Laboratory test data comprised white blood cell count, neutrophil count, lymphocyte count, monocyte count, red blood cell count, platelet count, red blood cell distribution width, and albumin level. Laboratory test data, collected within 24 h of hospital admission, included white blood cell count, neutrophil count, lymphocyte count, monocyte count, red blood cell count, platelet count, red blood cell distribution width, and serum albumin level. Clinical data encompassed preoperative diagnostic imaging results, detailed surgical records, and postoperative pathological reports.

### Definition of peripheral blood composite inflammatory markers

The following composite inflammatory markers were calculated based on peripheral blood test results, with all cell counts measured in *10^9^/L, except for red blood cell count in *10^12^/L, and red blood cell distribution width (RDW) in %, serum albumin level in g/L. NLR (12), Neutrophil-to-lymphocyte ratio; PLR (11), Platelet-to-lymphocyte ratio; LMR (13), Lymphocyte-to-monocyte ratio; SII (14), Systemic immune-inflammation index, calculated as platelet count * monocyte count/lymphocyte count; SIRI (10), Systemic inflammation response index, calculated as neutrophil count * monocyte count/lymphocyte count; PNI (15), Prognostic nutritional index, calculated as albumin level +5 * lymphocyte count; NAR (9), Neutrophil-to-albumin ratio; PAR (16), Platelet-to-albumin ratio; NPR (13), Neutrophil-to-platelet ratio; RLR (17), RDW-to-lymphocyte ratio.

### Statistical analysis

Statistical analyses were performed using R software (version 4.3.2). Continuous data were expressed as median (interquartile range, IQR:Q1, Q3). The Kolmogorov–Smirnov test was used to assess the normality of data distribution. For normally distributed continuous data, comparisons between the two groups were conducted using the independent samples *t*-test, and for non-normally distributed continuous data, the Wilcoxon rank-sum test was applied. Count data were described as *n* (%), and intergroup comparisons were performed using the Pearson chi-square test. The optimal cut-off values for the continuous variables were calculated using the “logrank” function from the “cutoff” package in R. In the training set, univariate logistic regression analysis was utilized to explore factors distinguishing CAA from SAA. Variables with a *P*-value <0.1 in univariate analysis were included in the subsequent multivariate stepwise logistic regression analysis. The stepwise selection was based on the Akaike Information Criterion (AIC), with the model having the lowest AIC selected as the optimal model to reduce multicollinearity. The variance inflation factor (VIF) was used to quantify multicollinearity among independent variables, a VIF <10 indicated no significant multicollinearity. A nomogram was constructed based on the final multivariate logistic regression model to visualize the combined prediction model. The diagnostic performance of each independent predictor and the nomogram model for differentiating CAA from SAA was evaluated using receiver operating characteristic (ROC) curves, with the area under the curve (AUC). The calibration of the nomogram was assessed using a calibration curve generated via 1000 bootstrap resamples, and the consistency between predicted probabilities and actual outcomes was quantified using the Hosmer-Lemeshow test. Decision curve analysis (DCA) was performed to evaluate the clinical utility of the nomogram. The validation set was used to further validate the actual performance of the model. Additionally, 10-fold cross-validation was performed on the full dataset to further evaluate the stability and generalizability of the prediction model. All statistical tests were two-sided, with a significance level set at *α* = 0.05. A *P*-value <0.05 was considered statistically significant.

### Ethics approval and consent to participate

This study was approved by the Ethics Committee of the 906th Hospital of Joint Logistic Support Force of PLA (Ethics Approval Number: PLA906-Research-20250806). All patients signed informed consent documentation before their inclusion in the study and all the procedures followed the ethical standards of the World Medical Association Declaration of Helsinki.

## Results

### Baseline characteristics of the study population

**Figure 1:**
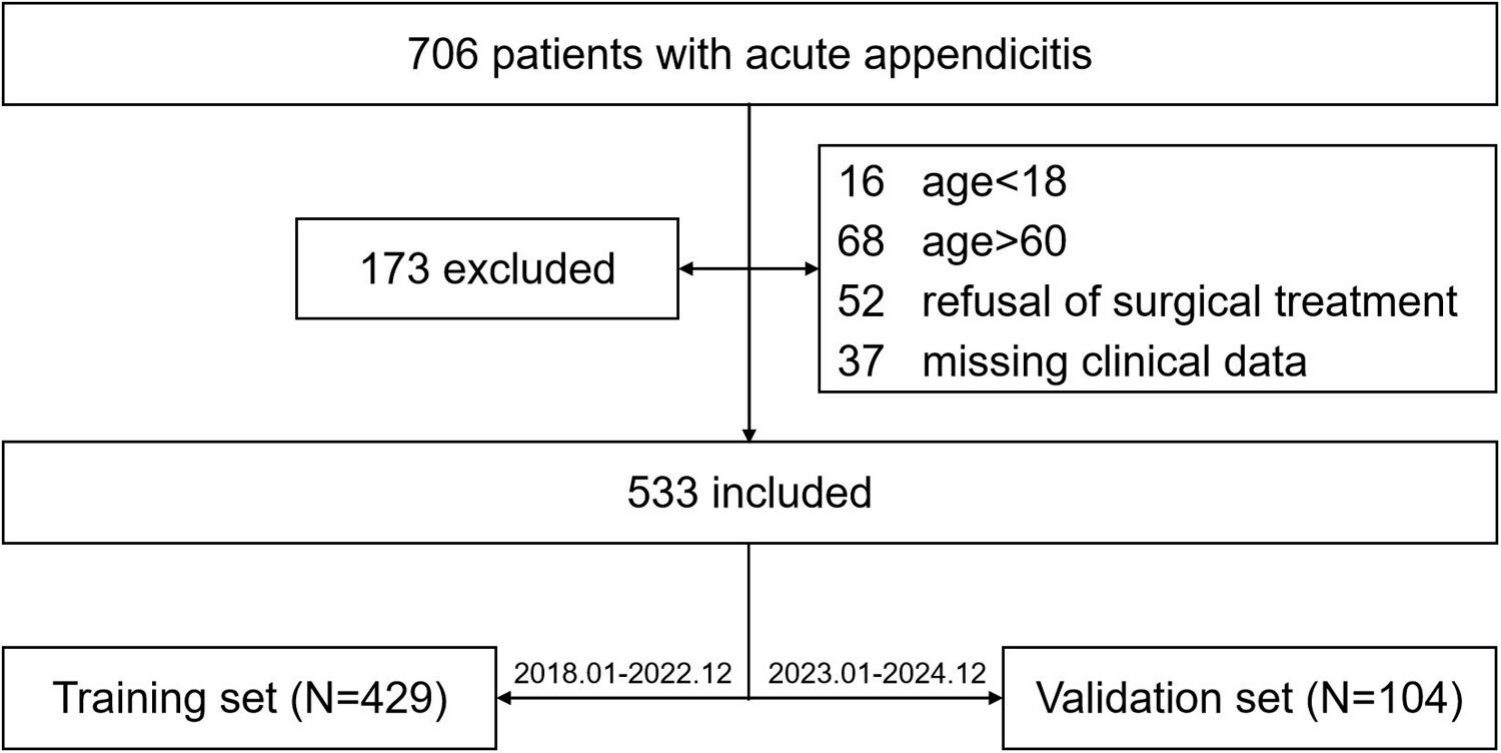
Participant flow in the study.

A total of 706 patients were initially considered for this study. Among them, 16 patients were under 18 years of age, 68 patients were over 60 years of age, 52 patients declined surgical intervention, and 37 patients had incomplete clinical data. Consequently, 533 patients who met the inclusion and exclusion criteria were ultimately enrolled (full dataset, 429 in the training set and 104 in the validation set, Figure 1). Table 1 presents the differences between patients in the training set and validation set. No significant differences were observed in age, history of smoking, or history of smoking between the two groups. However, the proportion of males in the training set was higher than that in the validation set (72.5% vs. 56.7%, *P* = 0.028), and there was a significant difference in the incidence of complicated acute appendicitis between the two groups (59.0% vs. 22.1%, *P* < 0.001). In addition, significant differences were noted in peripheral blood indices and composite inflammatory markers between the two groups (all *P* < 0.05), except for red blood cell distribution width (RDW) and platelet (PLT).

**Table 1:** Baseline characteristics of the study population .

Variable	Training set (*N* = 429)	Validation set (*N* = 104)	*P* value
Age (years)	26 (22, 37)	30 (20, 36)	0.924
Gender/Male	311 (72.5%)	59 (56.7%)	0.028
Type/CAA	253 (59.0%)	23 (22.1%)	<0.001
WBC (*10^9^/L)	9.6 (6.3, 13.57)	6.19 (5.08, 8.54)	<0.001
Neutrophil (*10^9^/L)	7.4 (3.97, 11.3)	4.12 (3.09, 6.68)	<0.001
Lymphocyte (*10^9^/L)	1.5 (1.11, 1.91)	1.45 (1, 1.65)	0.019
Monocyte (*10^9^/L)	0.5 (0.34, 0.72)	0.35 (0.25, 0.52)	<0.001
RBC (*10^12^/L)	4.76 (4.34, 5.05)	4.48 (4.11, 4.74)	<0.001
RDW (%)	12.9 (12.5, 13.3)	13 (12, 14)	0.809
Platelet (*10^9^/L)	199 (169, 228)	205.5 (167, 247.25)	0.148
Albumin (g/L)	46.7 (43.4, 49.2)	40.6 (36.68, 44.15)	<0.001
History of smoking	123 (28.6%)	35 (33.6%)	0.380
History of alcohol	85 (19.8%)	23 (22.1%)	0.169
NLR	4.53 (2.29, 8.76)	3.01 (1.95, 5.28)	0.001
PLR	130.77 (100, 173.68)	152.95 (121.04, 206.46)	<0.001
LMR	3 (1.75, 4.9)	3.93 (2.19, 6.08)	0.003
PNI	54.4 (50.95, 57.8)	46.88 (43.16, 52.16)	<0.001
SII	67.44 (39.82, 110.13)	51.79 (29.78, 100.86)	0.033
SIRI	2.43 (0.8, 5.89)	1.07 (0.49, 2.69)	<0.001
NAR	0.16 (0.09, 0.24)	0.11 (0.07, 0.18)	<0.001
PAR	4.25 (3.64, 4.95)	4.99 (4.19, 6.28)	<0.001
NPR	0.04 (0.02, 0.06)	0.02 (0.01, 0.03)	<0.001
RLR	8.53 (6.6, 11.73)	9.30 (7.57, 12.80)	0.012

### Baseline characteristics of patients in the training set

The training cohort comprised 311 males (72.5%) and 118 females (27.5%), with a median age of 26 years (IQR: 22, 37). A history of smoking was reported in 123 patients (28.7%), and alcohol consumption was reported in 85 patients (19.8%). Based on postoperative pathological reports, patients were categorized into two groups: 176 patients (41.0%) with simple acute appendicitis (SAA group) and 253 patients (59.0%) with complicated acute appendicitis (CAA group). All patients were successfully treated. Comparison of preoperative baseline characteristics between the two groups revealed no significant differences in age, gender distribution, proportion of smokers, or proportion of alcohol consumers (*P* > 0.05). However, white blood cell (WBC), neutrophil (Neu), and monocyte (Mon) counts were significantly lower in SAA group compared to CAA group, while the lymphocyte (Lym) count was slightly higher in SAA group. These differences were statistically significant (*P* < 0.001). In contrast, no significant differences were observed between the two groups in red blood cell (RBC) count, PLT, RDW, or albumin (ALB) levels (*P* > 0.05). Peripheral blood composite inflammatory indices were calculated according to the specified formulas. Comparative analysis demonstrated that the NLR, PLR, SII, SIRI, NAR, NPR, and RLR were significantly lower in SAA group than in CAA group. Conversely, the LMR was significantly higher in SAA group. All these differences were statistically significant (*P* < 0.001). No significant differences were found between the two groups for the PNI or PAR. Details are presented in Table 2.

**Table 2:** Baseline characteristics of patients in the training set.

Variable	SAA group (*N* = 176)	CAA group (*N* = 253)	*P* value
Age (years)	26 (22, 35)	27 (21, 39)	0.439
Gender/Male	123 (69.9%)	188 (74.3%)	0.369
WBC (*10^9^/L)	7.1 (5.64, 9.71)	12.08 (7.96, 14.87)	<0.001
Neutrophil (*10^9^/L)	4.69 (3.36, 7.74)	9.88 (6.15, 12.67)	<0.001
Lymphocyte (*10^9^/L)	1.64 (1.33, 2.09)	1.4 (1.04, 1.81)	<0.001
Monocyte (*10^9^/L)	0.42 (0.3, 0.6)	0.6 (0.4, 0.83)	<0.001
RBC (*10^12^/L)	4.75 (4.37, 5.06)	4.77 (4.33, 5.04)	0.762
RDW (%)	12.8 (12.5, 13.23)	12.9 (12.5, 13.3)	0.467
Platelet (*10^9^/L)	196 (169, 226)	200 (170, 229)	0.387
Albumin (g/L)	46.4 (43.3, 49.03)	47.1 (43.5, 49.6)	0.207
History of smoking	44 (25.0%)	79 (31.2%)	0.196
History of alcohol	30 (17.0%)	55 (21.7%)	0.282
NLR	2.88 (1.74, 4.65)	6.6 (3.65, 11.11)	<0.001
PLR	116.38 (91.33, 151.47)	140.83 (108.1, 190.74)	<0.001
LMR	4.17 (2.59, 5.8)	2.33 (1.54, 3.7)	<0.001
PNI	54.88 (51.4, 58.3)	54.35 (50.8, 57.5)	0.213
SII	47.88 (31.35, 79.96)	82.2 (51.79, 129.64)	<0.001
SIRI	1.19 (0.53, 2.52)	3.91 (1.83, 7.49)	<0.001
NAR	0.1 (0.07, 0.16)	0.21 (0.14, 0.27)	<0.001
PAR	4.18 (3.59, 4.9)	4.26 (3.66, 4.95)	0.871
NPR	0.03 (0.02, 0.04)	0.05 (0.03, 0.06)	<0.001
RLR	8.02 (6.12, 9.75)	9.07 (7.06, 12.65)	<0.001

### Risk factors for differentiating simple and complicated acute appendicitis

The optimal cutoff values for continuous variables were determined using R software, and these continuous variables were then converted into binary categorical variables based on the identified cutoffs (detailed cutoff values and categorization criteria are presented in Supplementary Table S1). All categorical variables were included in univariate logistic regression analysis to screen for factors associated with distinguishing CAA from SAA (Table 3). Variables with a *P*-value <0.1 in the univariate analysis were further included in multivariate stepwise logistic regression analysis. Using the backward stepwise method, variables that resulted in the largest increase in the AIC were sequentially removed from the full model until the optimal AIC was achieved (Supplementary Table S2). The final multivariate logistic regression results demonstrated that an elevated NAR (OR = 3.099, 95% CI: 1.693–5.723, *P* < 0.001), an elevated SIRI (OR = 2.972, 95% CI: 1.722–5.187, *P* < 0.001), and a decreased PNI (OR = 0.553, 95% CI: 0.309–0.969, *P* = 0.041) were independent risk factors for distinguishing CAA from SAA (Table 4). Additionally, VIF was used to assess the degree of multicollinearity among the three risk factors. All VIF values were less than 3 (2.434, 1.750 and 2.186, respectively), indicating no significant multicollinearity among them.

**Table 3:** Univariate logistic regression analysis for discriminating simple and complicated acute appendicitis.

Variable	OR	95% CI	*P* value
Age (years)	1.363	0.922–2.015	0.120
Gender/Male	1.246	0.811–1.911	0.313
WBC (*10^9^/L)	5.799	3.798–8.992	<0.001
Neutrophil (*10^9^/L)	6.04	3.922–9.472	<0.001
Lymphocyte (*10^9^/L)	0.42	0.273–0.639	<0.001
Monocyte (*10^9^/L)	2.988	1.994–4.524	<0.001
RBC (*10^12^/L)	1.194	0.807–1.767	0.375
RDW (%)	1.284	0.872–1.895	0.207
Platelet (*10^9^/L)	1.458	0.986–2.16	0.059
Albumin (g/L)	1.505	1.023–2.221	0.038
NLR	6.362	4.135–9.964	<0.001
PLR	2.295	1.549–3.421	<0.001
LMR	0.251	0.167–0.376	<0.001
PNI	0.697	0.471–1.028	0.069
SII	4.19	2.794–6.341	<0.001
SIRI	6.45	4.231–9.974	<0.001
NAR	6.947	4.36–11.399	<0.001
PAR	1.225	0.833–1.802	0.302
NPR	5.161	3.393–7.967	<0.001
RLR	2.412	1.575–3.745	<0.001
History of smoking	1.362	0.887–2.11	0.161
History of alcohol	1.352	0.83–2.235	0.231

**Table 4:** Multivariate stepwise logistic regression analysis for discriminating simple and complicated acute appendicitis.

Variable	OR	95% CI	*P* value
Albumin (g/L)	1.726	0.985–3.086	0.061
PNI	0.553	0.309–0.969	0.041
SIRI	2.972	1.722–5.187	<0.001
NAR	3.099	1.693–5.723	<0.001

### Construction and efficacy evaluation of the diagnostic model for differentiating simple and complicated acute appendicitis

**Figure 2:**
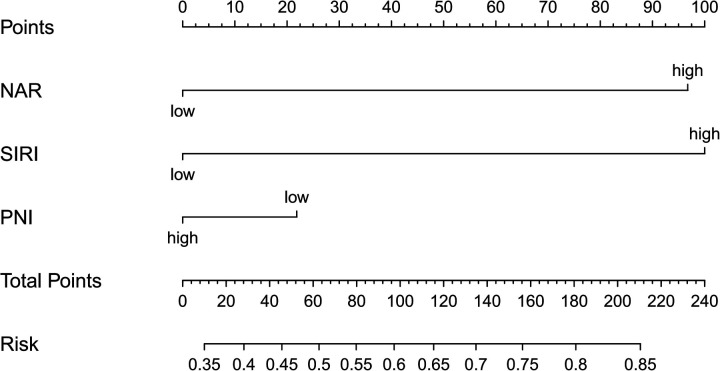
Nomogram predicting complicated acute appendicitis.

**Figure 3:**
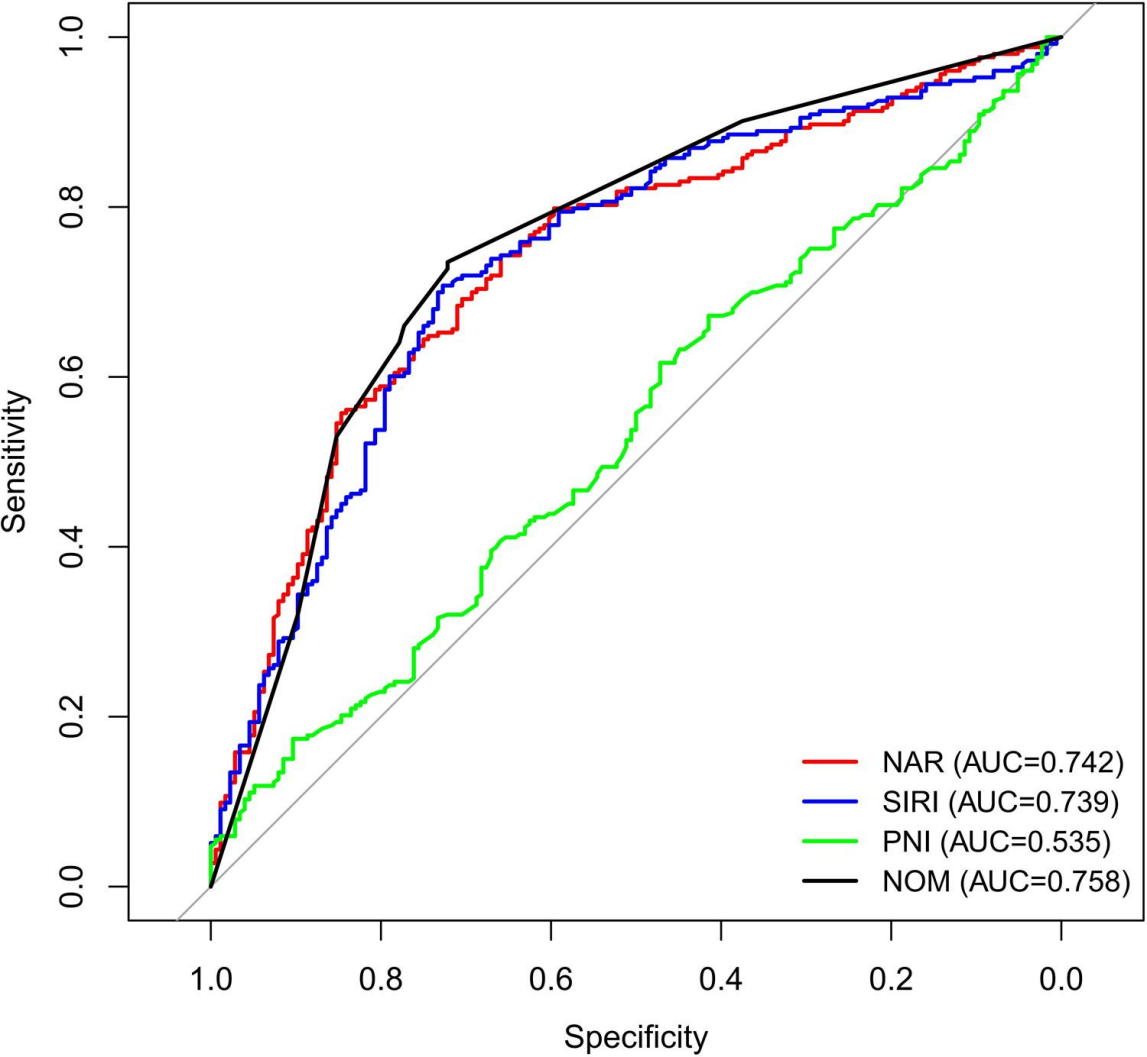
ROC of risk factors and nomogram.

**Figure 4:**
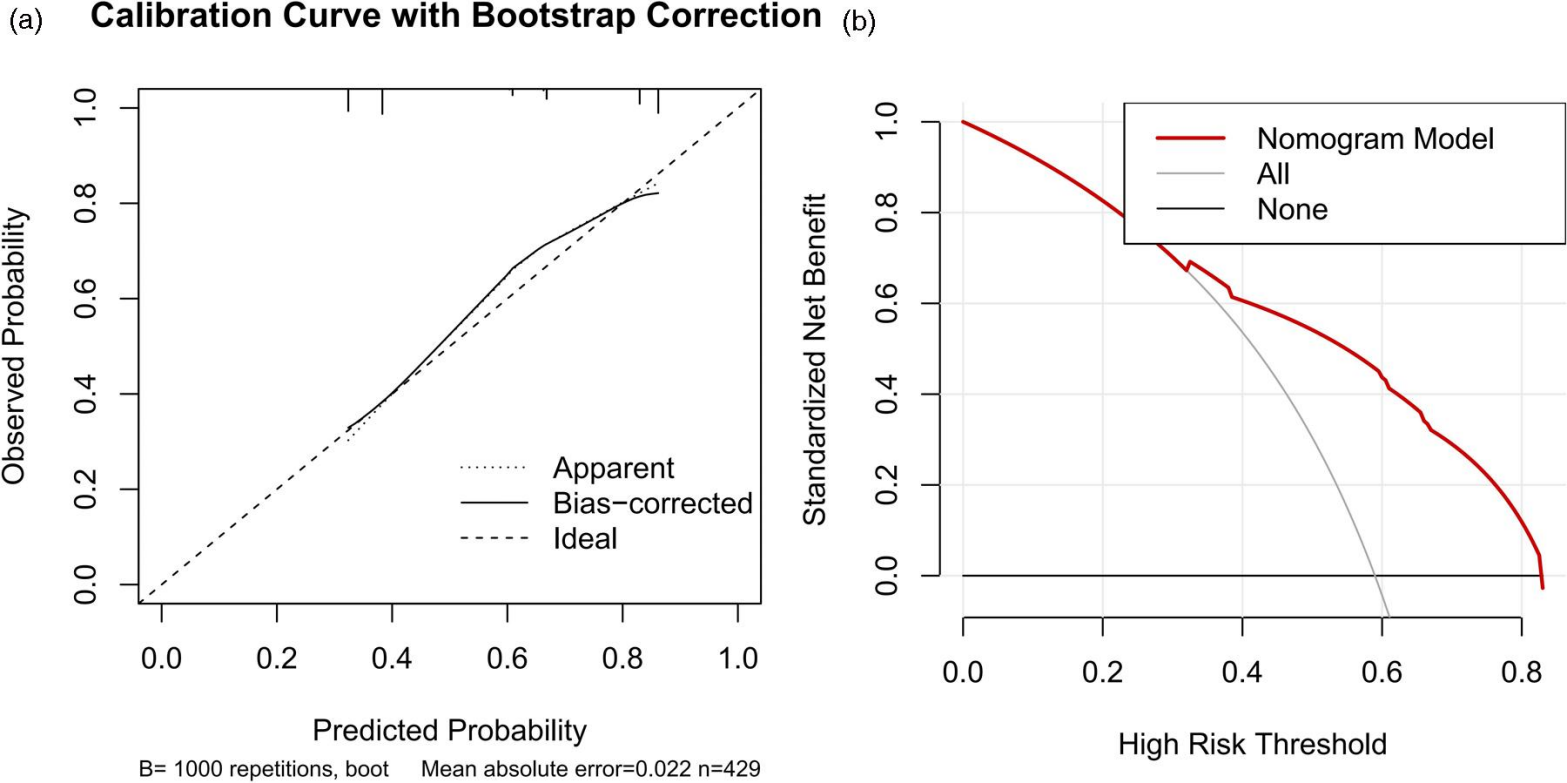
Calibration curve and decision curve analysis of the nomogram. **(A)** Bootstrap-based calibration curve of the nomogram; **(B)** Decision curve analysis based on the nomogram.

Based on the results of the multivariate stepwise logistic regression analysis for differentiating simple from complicated acute appendicitis, a prediction model was constructed using the nomogram method (Figure 2). The discriminatory efficacy of each independent risk factor and the nomogram model was subsequently analyzed using ROC curves. As shown in Figure 3 and Table 5, among the independent influencing factors, the SIRI and the NAR demonstrated relatively good discriminatory diagnostic performance, with AUC of 0.739 (95% CI: 0.693–0.785) and 0.742 (95% CI: 0.696–0.787), respectively. In contrast, the PNI exhibited relatively weaker discriminatory ability, with an AUC of only 0.535 (95% CI: 0.480–0.591). Notably, the nomogram model constructed by combining the three predictors achieved a higher diagnostic efficacy, with an AUC of 0.758 (95% CI: 0.714–0.803). The Hosmer-Lemeshow test was used to assess the calibration of the nomogram model, and the result showed *P* = 0.872, indicating the good calibration. Furthermore, the bootstrap-based calibration curve of the nomogram model demonstrated excellent predictive performance, with a mean absolute error (MAE) of 0.022 (Figure 4A), suggesting that the predicted risk of CAA generated by the model is highly consistent with the actual pathological diagnosis. And the decision curve analysis (DCA) based on the nomogram indicated that within the clinically relevant threshold probability range of 40% to 80%, applying this model to guide clinical decisions yielded a higher net clinical benefit (Figure 4B).

**Table 5:** AUC of the risk factors and nomogram prediction model for predicting complicated acute appendicitis.

Variable	Cut-off	AUC	95% CI	Sensitivity	Specificity	*P* value
PNI	55.575	0.535	0.480–0.591	47.2%	61.7%	<0.001
SIRI	2.197	0.739	0.693–0.785	72.7%	70.8%	<0.001
NAR	0.195	0.742	0.696–0.787	84.7%	55.7%	<0.001
Nomogram	0.491	0.758	0.714–0.803	72.2%	73.5%	<0.001

### Validation of the nomogram model for differentiating simple and complicated acute appendicitis

**Figure 5:**
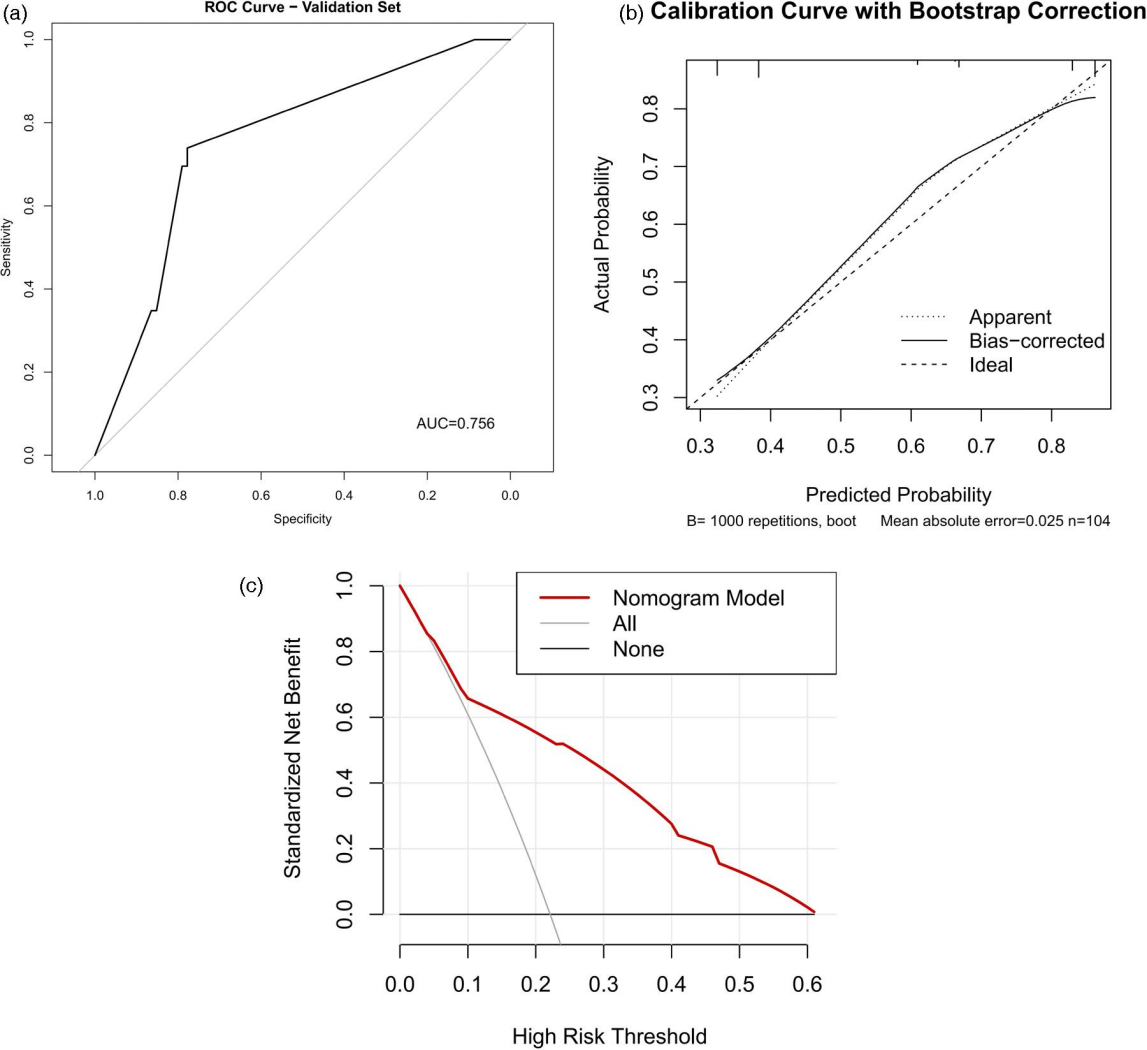
ROC curve, calibration curve and DCA curve of the nomogram in the validation set. **(A)** ROC curve of the nomogram for patients in the validation set; **(B)** Bootstrap-based calibration curve of the nomogram in the validation set; **(C)** Decision curve analysis based on the nomogram in the validation set.

**Figure 6:**
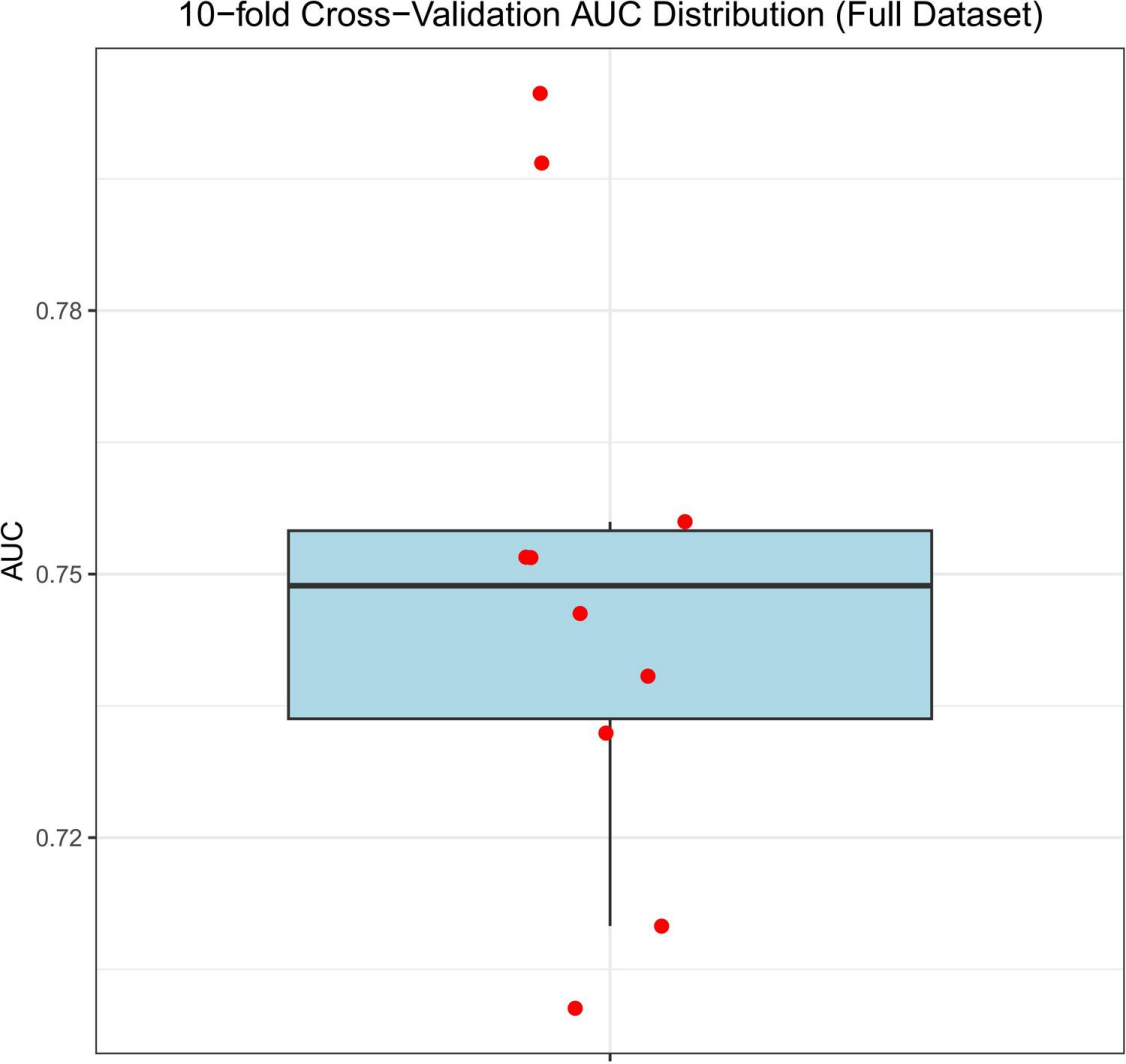
Boxplot of AUC values from 10-fold cross-validation on the full dataset.

Patients with acute appendicitis collected from January 2023 to December 2024 were assigned to the validation set, of whom 81 were in SAA group and 23 in CAA group. ROC curve analysis showed that the discriminative ability of the nomogram for complicated appendicitis in the validation set was basically consistent with that in the training set (Figure 5A, AUC = 0.756, 95%CI: 0.656–0.857). The calibration curve of the nomogram in the validation set demonstrated excellent predictive performance, with the MAE of 0.025 (Figure 5B). Meanwhile, the DCA in the validation set indicated that within the clinically relevant threshold probability range of 10% to 60%, the nomogram model additionally exhibited better performance than both the “treat all” and “treat none” strategies, highlighting its greater clinical applicability for supporting decision-making processes (Figure 5C). Furthermore, 10-fold cross-validation on the full dataset produced a median AUC of 0.749 (range: 0.701–0.805, Figure 6).

## Discussion

Acute appendicitis ranks as the fourth most common cause of acute abdominal pain (18), with a lifetime incidence of 8.6% in males and 6.9% in females (19). Accurate assessment of disease severity is crucial for clinical decision-making in the management of appendicitis. During the progression of appendicitis, significant alterations occur in the immune status and inflammatory levels of the body, suggesting that inflammatory markers may serve as potential indicators for distinguishing the severity of appendicitis. Currently, studies on inflammatory markers associated with complicated acute appendicitis suffer from limitations such as a narrow range of included markers and inconsistent findings. In this study, multiple peripheral blood composite inflammatory markers were incorporated, and it was ultimately determined that the NAR, SIRI, and PNI are independent predictors for differentiating between simple acute appendicitis and complicated acute appendicitis. Based on these predictors, a nomogram prediction model was constructed and validated to exhibit good discriminative diagnostic performance, thereby providing a valuable clinical reference for assessing the severity of acute appendicitis. Notably, the model relies on routine peripheral blood indices, making it low-cost, easily accessible, and suitable for widespread clinical application.

The results of this study indicate that among the composite inflammatory markers, SIRI, NLR, NAR, NPR, SII, LMR, PLR, RLR, and PNI are all potential factors for discriminating complicated acute appendicitis (13, 14, 16, 17, 20). In fact, numerous studies conducted in recent years on the association between composite inflammatory markers and acute appendicitis have demonstrated the discriminative ability of these aforementioned indicators. However, in the present study, only SIRI, NAR, and PNI were retained in the multiple stepwise logistic regression analysis. This highlights the research significance of selecting the optimal markers from a pool of promising inflammatory indicators.

In recent years, the significance of NAR as a biomarker in predicting the prognosis of severe inflammation has been widely discussed. A retrospective study involving 161 patients reported that NAR demonstrated good diagnostic value in distinguishing acute appendicitis from non-appendicitis diseases, supporting its utility as a composite inflammatory indicator in the diagnosis of acute appendicitis (9). However, it failed to predict acute perforated appendicitis. This limitation may be attributed to the fact that the study included only 22 patients with perforated appendicitis and did not enroll patients with other types of complicated appendicitis, such as suppurative appendicitis or gangrenous appendicitis. On the other hand, the study population included patients aged 10 to 94 years. Given that inflammatory responses differ between younger and older patients compared to adults (21), the presence of numerous confounding factors may have influenced the study results. In contrast, Saridas et al. found that NAR had a strong predictive ability for complicated acute appendicitis, with an AUC of 0.735 (22), which is largely consistent with the AUC of 0.742 for NAR observed in the present study.

A single-center study in Turkey involving 1,065 patients aged 0–18 years reported that 1,009 patients were diagnosed with acute appendicitis (10). The study demonstrated that the SIRI and SII exhibited strong diagnostic performance for acute appendicitis. When combined with other clinical indicators such as symptoms, signs, and imageological examinations, the diagnostic accuracy increased to 98%. In the present study, these additional indicators were not incorporated, which represents a limitation, however, this was consistent with the study's primary objective of evaluating the diagnostic utility of composite inflammatory markers. Nevertheless, that study did not find SIRI or SII to be capable of clearly distinguishing between complicated and non-complicated acute appendicitis.

Another investigation also reported favorable performance of SIRI and SII in diagnosing acute appendicitis (23). Moreover, both indices demonstrated moderate predictive value for complicated appendicitis, with AUC values of 0.634 for SII and 0.645 for SIRI. In the current study, the AUC of SIRI was 0.739. The discrepancy in AUC values may be attributable to patient heterogeneity. Additionally, SII was not identified as an independent risk factor for complicated acute appendicitis in our analysis. This difference may be explained by the fact that the aforementioned studies primarily compared AUC values of various parameters to determine the optimal indicator, whereas the present study employed multivariate stepwise logistic regression to identify independent risk factors. In support of our findings, Feier et al. performed multivariate logistic regression analysis on peripheral blood inflammatory markers in 407 patients with appendicitis and found that elevated SIRI was an independent predictor of acute phlegmonous appendicitis (OR = 2.615) and acute gangrenous appendicitis (OR = 2.970) (21). These results are largely consistent with those of the present study (OR = 2.972).

Both the NAR and SIRI involve neutrophils, indicating that neutrophils play a significant role in the pathogenesis of complicated appendicitis. Studies have shown that (24), when the appendiceal lumen becomes obstructed and bacterial overgrowth occurs, the immune system in the appendiceal tissue is extensively activated, and neutrophils are rapidly mobilized from the peripheral blood to the site of infection. However, excessive recruitment of neutrophils leading to their overactivation or dysfunction can exacerbate tissue damage, amplify the inflammatory response, and worsen the condition of appendicitis (25). Furthermore, monocytes and lymphocytes constitute the other two components of SIRI. Pathological studies have revealed significant monocyte infiltration in the appendiceal tissues of patients with acute appendicitis (26). Upon activation during inflammation, monocyte secrete substantial amounts of pro-inflammatory cytokines, such as IL-6 and TNF-α (27), which not only directly mediate tissue damage but also exacerbate the inflammatory response through cascade reactionse. Multiple studies have confirmed that serum and appendiceal tissue levels of IL-6 and TNF-α are closely associated with disease severity (e.g., perforation, gangrene) (28, 29). Concurrently, lymphocytes may decrease in acute inflammation due to overactivation or apoptosis, leading to an imbalance in adaptive immune responses (30). Additionally, lymphopenia may also be related to an infection-induced immunosuppressive microenvironment (31).

PNI is a composite indicator based on serum albumin levels and peripheral blood lymphocyte count, which assesses a patient's overall health status through nutritional status (albumin) and immune function (lymphocytes). It is commonly used to predict surgical risk and disease prognosis. In a study involving elderly patients with acute appendicitis, Kalayci et al. reported a strong association between PNI, albumin levels, and acute appendicitis (15). Albumin, a plasma protein synthesized by the liver, experiences reduced synthesis during acute inflammation due to the liver's preferential production of acute-phase proteins. Additionally, increased capillary permeability induced by inflammation leads to albumin extravasation, further decreasing serum levels (32). A study involving 62 patients with acute appendicitis found that an albumin level <2.85 g/dL was a key factor in distinguishing the severity of acute appendicitis, such as perforation or gangrene (33). This may be attributed to the significant suppression of albumin synthesis and enhanced extravasation when the degree of acute inflammation exceeds a certain threshold, thereby strengthening the association between albumin levels and disease severity (32). Furthermore, low albumin levels may reflect poor nutritional status and weakened immune function, which can facilitate the spread of appendiceal infection and increase the risk of suppuration, gangrene, and perforation. Therefore, the nutritional and immune status reflected by PNI may be indirectly associated with the inflammatory progression of acute appendicitis. In this study, PNI was identified as an independent predictor for differentiating between simple and complicated acute appendicitis. However, its AUC was only 0.535, indicating limited predictive performance, which may be attributed to the indirect rather than direct relationship between PNI and acute appendicitis.

Therefore, this study integrated the NAR, SIRI, and PNI indices to jointly construct a nomogram prediction model, which achieved an AUC of 0.758 (95% CI: 0.714–0.803). The Hosmer-Lemeshow test and calibration curves both indicated that the model possesses good predictive performance. And the DCA effectively delineates the threshold range where the nomogram model exhibits superior clinical utility in guiding decision-making. Additionally, we designated patients with acute appendicitis enrolled from January 2023 to December 2024 as the validation set. Notable differences in laboratory indicators and composite inflammatory markers were observed between the training and validation sets, which may be attributed to the significant discrepancy in the proportion of complicated acute appendicitis. This is because the division using the year 2023 as the cut-off point was not a random split, but rather a deliberate temporal demarcation based on a marked shift in clinical practice and healthcare-seeking behaviors associated with the Coronavirus Disease 2019 (COVID-19) pandemic. This time-based validation design makes the validation set a temporal validation cohort, which is not a methodological limitation but rather a more stringent test of the model's generalizability to assess its stability in an evolving clinical environment. The nomogram model maintained favorable predictive performance in the validation set, with an AUC of 0.756 (95%CI: 0.656–0.857). Furthermore, the calibration curve and DCA confirmed the accuracy of the nomogram model in identifying complicated acute appendicitis and its ability to guide clinical decision-making, demonstrating its good robustness and practical application value. Nevertheless, we performed 10-fold cross-validation on the full dataset and found a median AUC of 0.749 (range: 0.701–0.805). The consistency of AUC values across the training set, validation set, and cross-validation folds indicates that the model possesses stable discriminative ability and robust generalizability.

The nomogram developed in this study is designed to complement existing diagnostic pathways for suspected acute appendicitis. As its variables are routinely collected during initial emergency department assessment, the model can be implemented at the point of care once laboratory results are available, before advanced imaging is ordered. In patients with equivocal clinical presentations, a high nomogram-predicted probability of complicated acute appendicitis may lower the threshold for proceeding directly to abdominal computed tomography (CT), allowing earlier identification of those who may require urgent surgical intervention. By contrast, a low predicted probability in patients with mild symptoms could support a conservative approach involving clinical observation or initial ultrasound imaging, thereby reducing unnecessary radiation exposure and healthcare costs. Moreover, in resource-limited settings with limited or no access to CT, the nomogram provides an objective, quantitative tool to assist clinical decision-making when triaging patients for early surgical consultation vs. conservative management. Importantly, the nomogram should not be used in isolation; its results must be interpreted alongside clinical examination findings and, where available, ultrasound imaging.

However, this study has certain limitations. First, due to its single-center, retrospective nature, further prospective, multi-center studies with large sample sizes are required to validate the generalizability and feasibility of the conclusions. Additionally, due to the retrospective design of this study and the absence of systematically documented physical examination findings (e.g., rebound tenderness, anorexia) in the medical records, we were unable to combine our composite inflammatory markers with clinical symptoms and signs to construct a more accurate, multidimensional predictive model. This remains a goal for our future prospective studies.

In summary, this study investigated the value of peripheral blood composite inflammatory markers in differentiating between patients with simple and complicated acute appendicitis. It confirmed the close relationship of NAR, SIRI, and PNI with the severity of appendicitis, and subsequently developed a nomogram model with favorable diagnostic performance. This provides an important reference for clinicians to accurately assess patient conditions and formulate individualized treatment plans.

## Data Availability

The raw data supporting the conclusions of this article will be made available by the authors, without undue reservation.
