# Supplementary material for: Development and validation of a diagnostic nomogram based on peripheral blood composite inflammatory markers for complicated acute appendicitis: a retrospective study
Source: Front Surg. 2026 Apr 30;13:1784195. doi: 10.3389/fsurg.2026.1784195 (PMC13171817; doi:10.3389/fsurg.2026.1784195)
Supplement: Supplementary file 1 [file Table1.docx]

**SUPPLEMENTARY TABLE S1 |** Assignment table for converting continuous variables into categorical variables based on the optimal cut-off values obtained by R software

| Variable | Cut-off value | Convert to categorical variable |
| --- | --- | --- |
| Age (years) | 24.5 | low:<24.5, high:≥24.5 |
| WBC (*10^9^/L) | 9.76 | low:<9.76,  high:≥9.76 |
| Neutrophil (*10^9^/L) | 8.18 | low:<8.18,  high:≥8.18 |
| Lymphocyte (*10^9^/L) | 1.305 | low:<1.305,  high:≥1.305 |
| Monocyte (*10^9^/L) | 0.545 | low:<0.545,  high:≥0.545 |
| RBC (*10^12^/L) | 4.635 | low:<4.635,  high:≥4.635 |
| RDW (%) | 12.95 | low:<12.95,  high:≥12.95 |
| Platelet (*10^9^/L) | 186.5 | low:<186.5,  high:≥186.5 |
| Albumin (g/L) | 46.85 | low:<46.85,  high:≥46.85 |
| NLR | 4.842 | low:<4.842,  high:≥4.842 |
| PLR | 144.804 | low:<144.804,  high:≥144.804 |
| LMR | 3.271 | low:<3.271,  high:≥3.271 |
| PNI | 55.575 | low:<55.575,  high:≥55.575 |
| SII | 57.305 | low:<57.305,  high:≥57.305 |
| SIRI | 2.197 | low:<2.197,  high:≥2.197 |
| NAR | 0.195 | low:<0.195,  high:≥0.195 |
| PAR | 4.206 | low:<4.206,  high:≥4.206 |
| NPR | 0.041 | low:<0.041,  high:≥0.041 |
| RLR | 9.921 | low:<9.921,  high:≥9.921 |

**SUPPLEMENTARY TABLE S2 |** The optimal AIC through multivariate stepwise logistic regression analysis

| AIC | Multivariate stepwise regression analysis model |
| --- | --- |
| 507.05 | SIRI + NLR + Neu + WBC + NAR + NPR + SII +  LMR + Mon + PLR + Lym + RLR + ALB + PLT + PNI |
| 505.06 | SIRI + NLR + Neu + WBC + NAR + SII + LMR + Mon + PLR + Lym + RLR + ALB + PLT + PNI |
| 503.09 | SIRI + NLR + Neu + WBC + NAR + SII + LMR + PLR + Lym + RLR + ALB + PLT + PNI |
| 501.23 | SIRI + NLR + Neu + WBC + NAR + SII + PLR + Lym + RLR + ALB + PLT + PNI |
| 499.43 | SIRI + NLR + Neu + WBC + NAR + SII + PLR + Lym + ALB + PLT + PNI |
| 497.56 | SIRI + NLR + Neu + WBC + NAR + SII + PLR + ALB + PLT + PNI |
| 495.99 | SIRI + NLR + WBC + NAR + SII + PLR + ALB + PLT + PNI |
| 494.46 | SIRI + NLR + WBC + NAR + SII + ALB + PLT + PNI |
| 492.99 | SIRI + NLR + WBC + NAR + ALB + PLT + PNI |
| 491.83 | SIRI + NLR + NAR + ALB + PLT + PNI |
| 491.29 | SIRI + NAR + ALB + PLT + PNI |
| 490.87 | SIRI + NAR + ALB + PNI |

 Neu, Neutrophil; Mon, Monocyte; Lym, Lymphocyte; PLT, Platelet; ALB,Albumin.
